# Extraordinary Case of Protracted Vomiting, in Which Life Was Prolonged for an Unusual Length of Time without Food

**Published:** 1832

**Authors:** Daniel Sexton

**Affiliations:** New Harmony, Indiana


**Art. II.**—*Extraordinary Case of Protracted Vomiting, in which Life was sustained for an unusual length of time without Food.* Communicated by Daniel Sexton, M. D., of New Harmony,

I was requested on the 17th of October, 1829, to visit Mrs. L. L., who had been for some time declining in health. For five weeks she had been confined to her bed, vomiting frequently, and unable to retain any nourishment, life having been preserved in the mean time, by the occasional administration of a nutritive enema. She was much emaciated, although the countenance retained considerable vivacity. The pulse was weak, but without much disturbance, the skin of a natural temperature.

To allay the vomiting, I advised the following combination:

Aqua Ammoniæ,

Laudanum aa. ozi.

Oil of Cinnamon, gtt. viii.

Fifteen drops on a lump of loaf sugar, to be taken into the mouth and swallowed gradually. For a short time after taking the first dose, she expressed great satisfaction at the relief it produced, but on visiting her a few hours afterward, she informed me like every thing else she took, it had been followed by distressing vomiting, and had produced a great gastric distress.

Having ascertained from her husband that a few teaspoonfuls of brandy which she had taken some days before, had produced temporaay relief, and remained longer upon the stomach than any thing else she had taken, I recommended it to be tried in larger quantities, and in conjunction with it to rub the spine with laudanum. and apply an opium plaster to the stomach. This plan was adopted, and continued until the morning of the 19th, when it was ascertained that no reliance could be placed upon it.

She was now much worse, mouth dry, tongue covered with dark crust, and the skin warmer than natural, and her life was despaired of by all that saw her. I now proposed to her husband the use of crude mercury, and with his assent gave an ounce of it, apparently, with an immediate good effect. A second dose was administered in the course of the day, the good effect of which was not so apparent. The quick silver came away by stool, in minute globules, in the course of three or four days.

Several other remedies were tried in the course of this and the following day, every thing failing to give more than a temporary relief to the vomiting, and her system still sinking. The Spts. of Turpentine, in doses of a dram, mixed with mucilage, appeared for a time to relieve her, astonishingly, but soon failed to produce any influence. Supposing her to be dying, at 2 o'clock in the morning of the 21st, I gave her 1 1-2 grains of opium, and left without the expectation of again seeing her alive. However, to my great surprise, on the morning of the next day, she was found to be much better. In the evening she took an enema which brought away dark fœtid evacuations, and from this time she began to recover rapidly, and by the 23d, considered herself out of danger.

She had now lived for six weeks without food, and her body formed the most complete skeleton that could be conceived consistently with the continuance of life, the limbs appearing to be held together by the ligaments and integuments alone. Her intellectual faculties, however, were unimpaired, and the love of life undiminished.

We now gave her porter, wine, and sago in small quantities, with, occasionally, a little chicken soup thickened with barley. Upon this diet she continued to recover until the 3d of November. At this time it became necessary to remove her to the house of a neighbor, in consequence of the indisposition of her husband rendering him unable to attend upon her. She was borne upon a litter, supported by four men, when the unusual motion brought on a return of the vomiting, which continued for three days at protracted intervals. At the expiration of this time, in consequence of some imprudence in diet, it returned with great violence, threatening to prostrate her immediately.

Other measures having been ineffectual in relieving her, I resorted to the use of belladonna. One grain divided into four pills, one of which to be taken every six hours. These were continued until five grains were taken, with the effect of keeping the stomach perfectly composed. At the expiration of this time the belladonna began to manifest its constitutional effects upon the system, it was discontinued for 36 hours, when upon some indications of a return of the vomiting, two additional doses of the belladonna were taken, which relieved it entirely.

From this time her recovery was slow and gradual, but uninterrupted. Some difficulty was experienced in restoring a regular condition of the bowels, but by the aid of injections and laxatives this was brought about, and a healthy state of all the secretions established.

About the first of December, after sitting up so long as to produce fatigue, she complained of a disagreeable sensation of pricking over the body, similar to that arising from pressure upon a nerve, and during the two or three days following, this sensation increased to such a degree of intensity in the hands and feet, as occasionally, to produce a temporary delirium. It was very much mitigated by the use of cider and water, but, for many days it returned about two hours after eating, accompanied with an unpleasant sense of burning. A local application of brandy to the hands and feet gave very great relief.

By spring, her health and strength were perfectly restored, except some inability of using the lower extremities, which gradually yielded to a system of regular exercise.

